# Enhancing Force Absorption, Stress–Strain and Thermal Properties of Weft-Knitted Inlay Spacer Fabric Structures for Apparel Applications

**DOI:** 10.3390/polym16213031

**Published:** 2024-10-29

**Authors:** Mei-Ying Kwan, Yi-Fan Tu, Kit-Lun Yick, Joanne Yip, Nga Wun Li, Annie Yu, Ka-Wai Lo

**Affiliations:** 1 School of Fashion and Textiles, The Hong Kong Polytechnic University, Hong Kong, China; 2 Faculty of Design, Architecture and Building, University of Technology Sydney, Sydney, NSW 2007, Australia

**Keywords:** cushioning material, compression evaluation, inlay knitting, stretchability, sustainability

## Abstract

The pursuit of materials that offer both wear comfort and protection for functional and protective clothing has led to the exploration of weft-knitted spacer structures. Traditional cushioning materials such as spacer fabrics and laminated foam often suffer from deformation under compression stresses, thus compromising their protective properties_._ This study investigates the enhancement of the force absorption, stress–strain, and thermal properties of weft-knitted spacer fabrics with inlays. Surface yarns with superior stretchability and thermal properties are used and combined with elastic yarns in various patterns to fabricate nine different inlay samples. The mechanical and thermal properties of these samples are systematically analyzed, including their compression, stretchability, thermal comfort, and surface properties. The results show that the inlay spacer fabric exhibits superior compression properties and thermal conductivity compared to traditional laminated foam and spacer fabrics while maintaining stretchability, thus providing better performance than traditional fabrics for protective clothing and wearable cushioning products. This study further confirms that the type of inlay yarn and inlay structure are crucial factors that significantly influence the thermal, tensile, and compressive properties of the fabric. This research provides valuable insights into the design and development of advanced textile structures to improve wear comfort and protection in close-fitting apparel applications.

## 1. Introduction

Exploration of materials that provide both wear comfort and protection has long been a challenge in the design and development of functional and protective apparel products. Traditional cushioning materials such as spacer fabrics and foam materials have been integral in providing the necessary protection and wear comfort for various applications, which range from insoles, helmet liners, wound dressings, and sports bras [1,2,3,4,5,6,7]. These materials possess favorable compression characteristics, adequate thickness, and the ability to serve as protective layers, especially around the bony prominences. Spacer fabrics consist of a connective layer formed by spacer filaments, which are created by using tuck stitches with both the front and back needle beds, thereby supporting the two layers of surface fabric [8]. This sandwich-like structure gives spacer fabrics excellent air and moisture permeabilities, cushioning performance, and pressure distribution [9,10,11]. Similarly, foam materials, including the innovative auxetic foam, are gaining traction in garment manufacturing due to their versatile properties and potential applications, including padded clothing and footwear [12]. The porosity of foam sheets can be tailored to customize physical properties such as impact response [13,14,15].

Despite their widespread use, both spacer fabrics and foam materials are prone to deformation, particularly when subjected to high compression stress from body weight. This stress can lead to the failure of the interlacing yarns and fabric structures, thus resulting in a reduction in the cushioning properties. Thin weft-knitted spacer fabrics are likely to collapse under such stress, which negatively impacts their energy absorption, protective capability, and shape retention in apparel applications. To address these challenges, the incorporation of inlays during knitting has been proposed to reinforce these fabrics and enhance their mechanical properties. Inlay yarns, incorporated through miss or tuck stitches during knitting, have been shown to improve fabric stability, stretchability, elastic recovery, compression properties, hand feel, and aesthetics of the knitted structure [16,17].

Yu et al. [18] successfully used elastic yarns and high-performance materials such as aramid and fiberglass as inlay materials in the fabrication of socks, gloves, and composites. Lu et al. [19] inserted inlay yarns and laid-in stitches to effectively control fabric tension behavior and pressure performance. The inlay fabric is stronger and resistant to breakage in the course direction when the diameter of the inlay material is increased [20]. When silicone and foam tubes are inlaid into the connective layer, the fabric structure offers superior compression resistance and absorption against impact forces. The correlation between Young’s modulus of inlay silicone tubes and fabric compression behavior has been explored [21]. When foam rods are used as the inlay yarn for cushioning insoles, the increased inlay density and spacer yarns show a significant influence in reducing and absorbing the impact forces.

However, the addition of extra yarns or foam tubes to enhance reinforcement and compression resistance can increase the rigidity of the material, so that it is less formable, thus restricting free body movement. The smaller span distance between the spacer and inlay yarns also impedes air and water vapor permeabilities, which largely limits the rate of heat exchange from the apparel to the ambient environment, and results in discomfort during wear. When utilized in protective clothing and next-to-skin apparel, it becomes crucial to strike a balance between providing adequate cushioning and support, to ensure flexibility for body movement, and maintaining a suitable thermal comfort.

Previous studies began to investigate inlay knitting technology and its applications, starting with the use of a V-bed hand knitting machine [20] and progressing to a 10-gauge flat-bed weft-knitting machine [18]. With the development of technology, this study proposes to use the 14-gauge WholeGarment knitting machine to insert finer yarns and speed up production, formulating fabrics with enhanced smoothness, stretchability, force absorption, and thermal properties. The choice of inlay yarns can also be upgraded from thick inlay tubes made of polyvinyl chloride and silicone polyethylene to soft and thin inlay yarns made of nylon or polyester, creating greater opportunities for material selection. Applications for inlay fabrics can also be used in close-fitting garments that require flexibility, smooth handfeel, and cushioning, such as bra cups.

The challenge extends to achieving the necessary levels of shape conformity and dynamic fit tailored to the movement of the body, which requires a highly complex design and production process, involving multiple engineering stages and specialized technologies. Fully fashioned knitting is a sustainable solution to address these challenges, which involves the direct knitting of garment panels from yarn, thereby reducing fabric waste, labor, and energy consumption associated with conventional manufacturing processes [22,23]. This method offers a variety of knitted structures and allows for the production of items with different shapes and functions, thus offering unlimited possibilities in garment design.

Despite advancements in cushioning materials, there remains a challenge in developing material structures that can retain stretchability while providing enhanced thermal comfort and compressive properties during daily activities. For example, many people are reluctant to wear protective work gloves as they limit hand dexterity, cause loss of finger sensation, and reduce work efficiency. In the engineering design of protective gloves, the use of stretchable fabric and protective paddings on the interphalangeal joints not only affects the ease of finger bending but is also associated with the displacement of the glove during hand movement.

It is known that the inlay elastic yarns can influence the curvature of weft-knitted spacer fabrics, thus contributing to the stretchability of fabrics for apparel products. However, the effects of inlay elastic yarns on the stress–strain behavior of spacer fabric structures have been largely neglected. It is anticipated that the type and nature of the inlay yarn, whether the yarn is rigid or elastic, plays a crucial role in changing the fabric characteristics, such as its stretch, compression, and force absorption properties. This study, therefore, provides a comprehensive analysis of the effects of the inlay structure and knitting parameters on the mechanical behavior of the resulting fabrics. The force absorption, stress–strain, and thermal properties of the inlay spacer fabrics are systematically analyzed and compared to those of conventional foam and spacer fabrics. The findings from this study are expected to significantly contribute to the design of close-fitting apparel with cushioning and protective features, such as orthopedic supports and sports bras.

## 2. Methodology

### 2.1. Surface Yarns

In this study, the experimental component comprises two stages. In Stage One, the surface yarns with the desired tensile elasticity and thermal properties for the fabrication of the inlay fabrics were chosen. In Stage Two, inlay fabric samples with different inlay orientations were fabricated.

Inlay fabric consists of surface yarns, inlay yarns, and spacer yarns. There are a variety of surface yarns available in the market. As shown in Table 1, seven types of surface yarns (namely, N1 to N7) are sourced commercially based on their inherent elasticity, softness, and compatibility with fully fashioned knitting techniques. To ensure the yarn quality for a smooth knitting process throughout the experiment, the yarns were first knitted into a single jersey fabric structure by using a 15L Shima Seiki SWG-XS WholeGarment knitting machine in 14-gauge (Wakayama, Japan). The fabric’s thermal conductivity, water vapor transmission (WVT), surface friction, and extension behavior were systematically investigated.

### 2.2. Inlay Spacer Fabric Samples

In Stage Two, two surface yarns identified in Stage One and chosen for their superior tensile and thermal properties, namely Surface Yarns A and B, were used for the fabrication of the inlay samples. These were combined with an elastic yarn and various inlay orientations were used. All of the inlay samples were produced by using the Shima Seiki WholeGarment knitting machine in Stage One [24].

A total of nine different inlay fabric samples were fabricated. The effects of the yarn composition and inlay structures on the tensile elasticity and thermal behavior of the fabric were thoroughly analyzed to determine the most suitable configuration for next-to-skin apparel applications. The fabrication details are summarized in Table 2 and Table 3. As shown in the table, the surface yarns are paired with a spandex/nylon blended yarn to improve their stretchability [25]. A 1/6 Nm 100% recycled stretch nylon yarn (Inlay Yarn X) and a 1/10 Nm 100% recycled polyester yarn (Inlay Yarn Y) were strategically selected to enhance the functionality of the knitted samples [26].

A total of three inlay orientations were adopted: Patterns I, II, and III. In Patterns I and III, a single layer of inlay yarns was inserted between the surface layers, whereas Pattern II has two layers of inlay yarns. To improve fabric stability and stretchability, KN70/70 Lycra was chosen as the spacer yarn for Patterns I and II, which enables the material to stretch and then return to its original shape [27]. Pattern III connects the fabric layers and secures the inlay yarns by crossing surface yarns between the front and back needle beds to provide sufficient thickness and strength for the final product.

**Figure 1:**
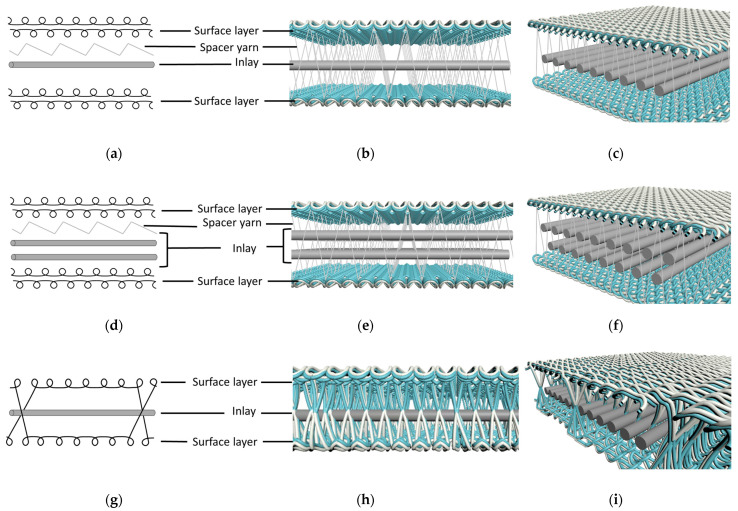
Schematic of inlay fabric: (**a**) knitting notation of Pattern I, (**b**) cross-sectional view of Pattern I, (**c**) close up of Pattern I, (**d**) knitting notation of Pattern II, (**e**) cross-sectional view of Pattern II, (**f**) close up of Pattern II, (**g**) knitting notation of Pattern III, (**h**) cross-sectional view of Pattern III, and (**i**) close up of Pattern III.

**Figure 2:**
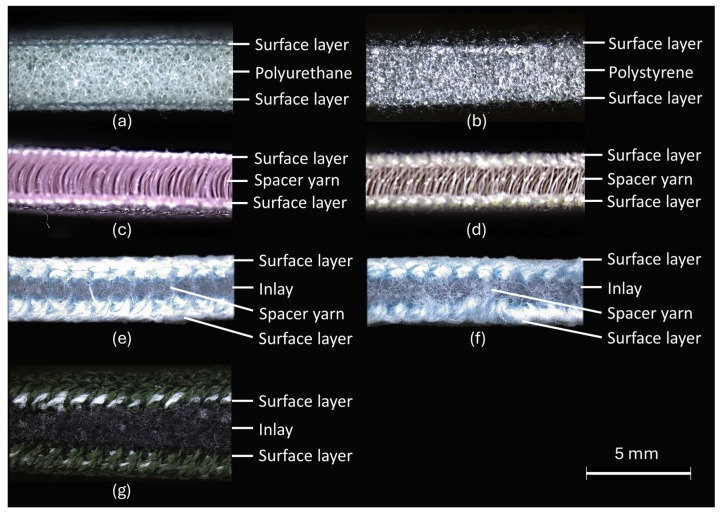
Microscopic cross-sectional view: (**a**) laminated foam F1, (**b**) laminated foam F2, (**c**) spacer fabric S1, (**d**) spacer fabric S2. (**e**) inlay spacer fabric with Pattern I, (**f**) inlay spacer fabric with Pattern II, and (**g**) inlay fabric with Pattern III.

**Figure 3:**
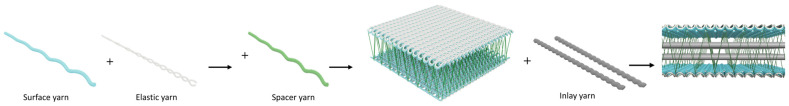
Production process of inlay fabric.

The physical properties of the inlay samples, including compression, stretchability, water vapor permeability, and thermal conductivity, were assessed. To understand the performance level of the inlay samples, the mechanical properties of inlay samples were compared with two typical laminated foams (F1 and F2), and two commercially available spacer fabrics (S1 and S2). Spacer fabrics and foam materials have been widely employed as cushioning materials, comparison with their properties can help evaluate the potential applications of inlay samples. The laminated foams have two surface layers of 100% polyester, where F1 is a layer of 100% polyurethane foam, while F2 is a layer of 100% polystyrene foam. Conversely, S1 comprises 92% polyester and 8% spandex, while S2 consists of 93% polyester and 7% spandex. The cross-sectional views of these fabrics are shown in Figure 1 and Figure 2. Taking fabric AXII as an example, Figure 3 further illustrates the production process of inlay samples.

### 2.3. Material Tests

In both stages of the study, three specimens were prepared for each fabric test. Laboratory tests were carried out in a controlled environment at a temperature of 20 ± 1 °C and a relative humidity of 65 ± 5% [18,20]. The test methods, standards, and devices utilized are summarized in Table 4.

The thermal properties, including thermal conductivity and WVT, were measured to evaluate the material comfort during wear. Thermal conductivity indicates heat conduction through the material, resulting in the amount of heat lost from the body to the environment. The equation considering material thickness and cross-sectional area is shown below

(1) $$k=\frac{q\times d}{A\times ∆T},$$

where:

k = thermal conductivity;

q = heat transfer rate (W);

A = cross-sectional area through which heat is being conducted (m^2^);

ΔT = temperature difference across the material (K);

d = thickness of the material (m).

WVT evaluates moisture permeability over a specific timeframe. According to the test standard specified in Table 4, it is calculated based on mass transfer over 1 day (i.e., 24 h), and testing area. The equation is shown below:

(2) $$WVT=\frac{M}{A\times t},$$

where:

M = mass of water vapor that passes through the material (g);

A = testing area (m^2^);

t = time period over which the measurement is taken (days).

The surface friction and roughness tests determine the unevenness or irregularities on the fabric surface and the resistance to friction of the fabric when sliding against another material, which is crucial for evaluating handfeel and wear comfort. The stretchability tests provide insights into the fabric elasticity and recovery, which are vital for garment fit and durability. Compression tests on the inlay fabric samples determine their structural integrity and support under pressure. The compression testing was conducted at a rate of 12 mm/min and the samples were compressed up to 80% of their initial thickness. In considering the protection of body from high impact forces, the force reduction performance of the fabric was also measured. The force reduction ability of a fabric refers to the percentage of the peak force that can be reduced, which is calculated by using:

(3) $$FRx=\left(\frac{1-Fx}{Fo}\right)\times 100\%\;,$$

where:

FRx = percentage of force reduction of a fabric sample (%);

Fx = peak force measured against the sample (N);

Fo = peak force measured against the ground (N).

### 2.4. Statistical Analysis

Statistical analysis was carried out by using the Statistical Package for the Social Sciences program (SPSS^®^21, IBM^®^ Corporation, New York, NY, USA). The one-way analysis of variance (ANOVA) was conducted to evaluate the performance differences between the inlay fabrics, laminated foam, and spacer fabrics, as well as the effects of different surface and inlay yarns, and patterns on the material properties of the inlay fabrics. Significance was set at 0.05.

## 3. Results and Discussion

### 3.1. Evaluation of Surface Yarn

In Stage One, the surface yarn was exclusively selected for its excellent thermal properties, handfeel, and stretchability. These are important elements when evaluating clothing that sits next to skin. The testing results of the seven types of surface yarns are shown in Table 5.

The WVT of N1 (33.0 g/m^2^/day) and N4 (36.5 g/m^2^/day) has higher values, thus indicating that they have good water vapor permeability, so that moisture can escape more easily, thus enhancing breathability and preventing moisture from accumulating on the skin. N1 also has the highest thermal conductivity among the samples (0.054 W/cm·°C). Fabrics with high thermal conductivity can transfer heat from the body to the external environment more effectively, which helps to maintain body temperature within a comfortable range, thus promoting thermal balance and preventing health problems caused by poor body heat control, such as hyperthermia [28]. Therefore, fabrics made of N1 are expected to have good thermal conductivity.

In terms of surface properties, N2 has lower values on average for the different directions and faces, which means that fabric produced by using N2 has lower friction and a smoother surface. Fabric smoothness is crucial to user experience and has a long-term impact on treatment compliance if the fabric is used for medical related purposes. In addition, fabrics constructed with N1 and N5 require particularly low loads to extend them to the specified elongation of 50%. Therefore, N1 and N5 enhance the stretchability of apparel products and allow free body movement during daily activities.

It is evident that surface yarn N1 shows excellent performance in thermal comfort and stretchability, while surface yarn N2 has the most desirable surface properties. In addition to excellent mechanical properties, N1 is made of an eco-conscious mix of 80% recycled viscose and 20% nylon, which is not only environmentally friendly but also imparts durability [29,30]. On the other hand, N2 features a blend of 60% organic cotton and 40% Renew Acetate, which enhances the breathability and eco-friendliness of the fabric [31,32], thus facilitating moisture management and increasing wear comfort during physical activities. Therefore, surface yarns N1 and N2 were selected for the development of the inlay fabrics in Stage Two. Spandex/nylon (15%/85%) blended yarn was added during production to further improve the tensile properties.

### 3.2. Evaluation of Knitted Fabrics with Inlays

In Stage Two where the inlay fabrics were knitted, N1 and N2 were adopted as Yarns A and B, respectively (Table 2). A total of nine inlay fabrics were then produced. The results of their physical properties in comparison with F1, F2, S1, and S2 are presented in this section. Table 6 further shows the effects of the different yarns and patterns on the material properties.

#### 3.2.1. Stretchability

**Figure 4:**
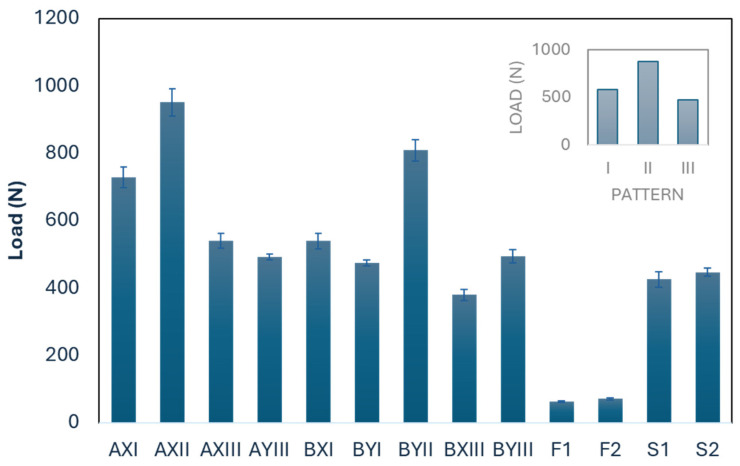
Results of tensile load for fabric elongation of 50% in the wale direction.

A comparative analysis of stretchability, as shown in Table 7 and Figure 4, reveals significant differences in the wale direction among the inlay fabric samples, and conventional laminated foam and spacer fabrics (*p* < 0.001). Among them, the peak load of laminated foam is much lower (F1: 63.7 N; F2: 70.9 N), thus indicating better stretchability. Yan et al. [33] indicated that the use of laminates in foam composites can improve tensile strength and tear resistance. As shown in Table 6, the pattern of the inlay fabric appears to significantly affect the tensile results (*p* = 0.067). In particular, Pattern III, characterized by crossing the surface yarns to hold one layer of inlay yarns, has the lowest energy requirement to achieve 50% elongation, that is, using a load of 476.4 N. This indicates that Pattern III has the highest level of stretchability among the tested patterns. Notably, its stretchability is comparable to that of conventional spacer fabrics. In contrast, Pattern II requires a significantly higher load (880.1 N) to achieve the same amount of elongation, which suggests that the insertion of spacer yarn and more than one layer of inlay yarns may have a detrimental effect on fabric stretchability. The results highlight the importance of fabric pattern designs in determining stretchability.

**Figure 5:**
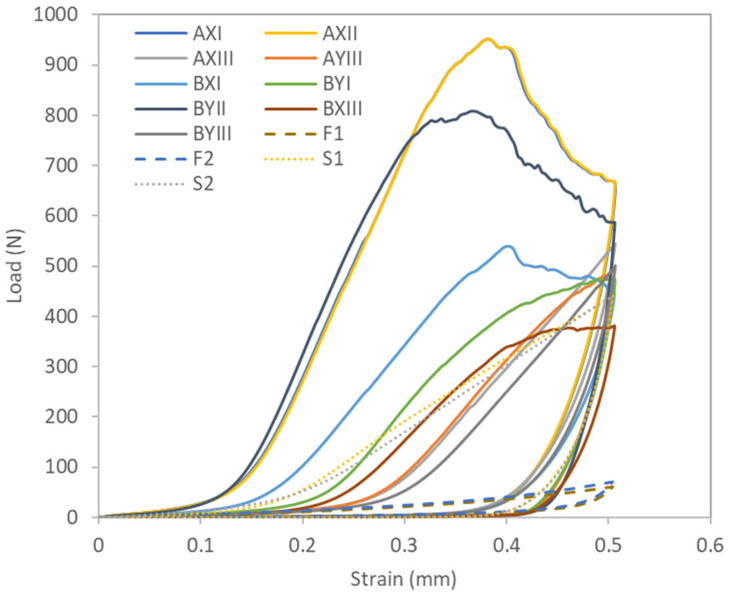
Load-strain curves in wale direction.

In addition, despite reaching higher peak loads, some of the inlay spacer fabrics, such as AXII and BYII, have a gradual decline post-peak, thus indicating that the fabrics are non-brittle and ductile, and can sustain some load even after initial failure (Figure 5). They will also revert back to their original shape upon the removal of the load, thus maintaining elasticity.

#### 3.2.2. Thermal Properties

The water vapor permeability test results did not show a significant difference between fabric types, which shows that our inlay spacer fabrics have similar water vapor permeability properties as conventional materials (Table 7). Li et al. [34] found that the WVT of a fabric can be changed by using different types of inlay yarns. Our study also shows that the inlay yarn has a certain effect on the results (*p* = 0.093), even though the effect is not statistically significant. The insignificant effect could be associated with the limitations of the knitting machine model (14 gauge), along with the selected thickness and rigidity of the inlay yarn used in the study.

**Figure 6:**
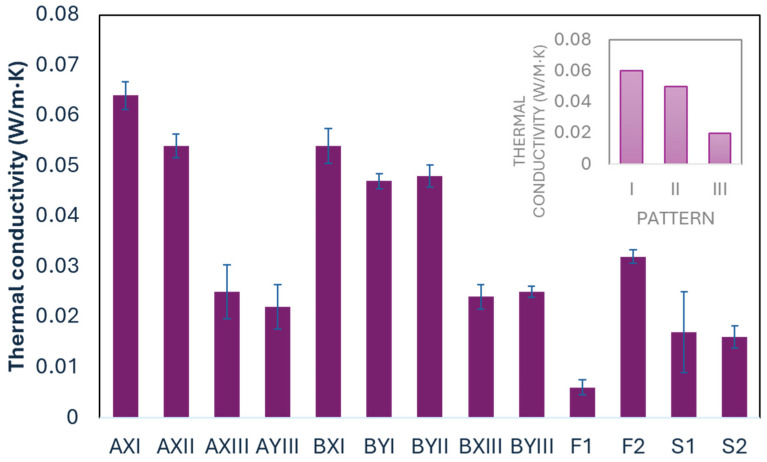
Results of thermal conductivity test.

The inlay spacer fabrics and commercially available laminated foam have a higher thermal conductivity than the spacer fabrics, with AXI having the highest thermal conductivity value (Table 7). This shows that the incorporation of inlay materials can significantly affect the thermal behavior of fabrics, potentially leading to improved heat transfer characteristics. Table 6 and Figure 6 further show that Pattern I has a higher thermal conductivity than Patterns II and III (*p* = 0.026). Therefore, more inlays and surface yarns used for tucks prevent heat transfer. Previous studies have also stated that the material composition plays an important role in influencing how inlay materials affect thermal isolation properties, and the thermal conductivity of fabrics can be affected by yarn type and fabric structure [35].

An analysis of fabric factors presented in Table 6 shows that the two selected surface yarns and the two proposed inlay yarns have similar effects on thermal conductivity. The combination of these yarns does not significantly affect the mechanical properties. Li et al. [34] found that the thermal conductivity of the inlay spacer structure can change with the diameter of the spacer yarn and the type of inlay yarn used.

Thermal performance is critical to providing comfort for the body. Materials with appropriate thermal conductivity can improve ventilation and promote heat dissipation. They can also allow moisture to escape from the skin surface, reduce moisture accumulation under clothing, enhance the evaporative cooling effect, and help prevent overheating. This is especially important in warm conditions or when engaging in physical activity.

#### 3.2.3. Compression Performance

Fabrics that exhibit superior performance under compression are more likely to provide enhanced wear comfort and support with longevity. A fabric that can withstand higher maximum compression forces means that it has a greater capacity to resist deformation under applied loads [34]. As shown in Table 7, the inlay spacer fabric samples show the ability to withstand higher maximum compression forces than the conventional laminated foam and spacer fabrics. Specifically, AXI can withstand the highest maximum compression force of 3028.2 N, followed by AXII of 1910.7 N. In contrast, the average maximum compression force that the laminated foam can withstand is 123.4 N, and spacer fabric is even lower at 58.9 N, which means that they are both more likely to deform under compression loading. Hence, the conventional materials tested are not suitable for garments or materials that require protective or cushioning functions, as they are prone to deform under applied forces.

**Figure 7:**
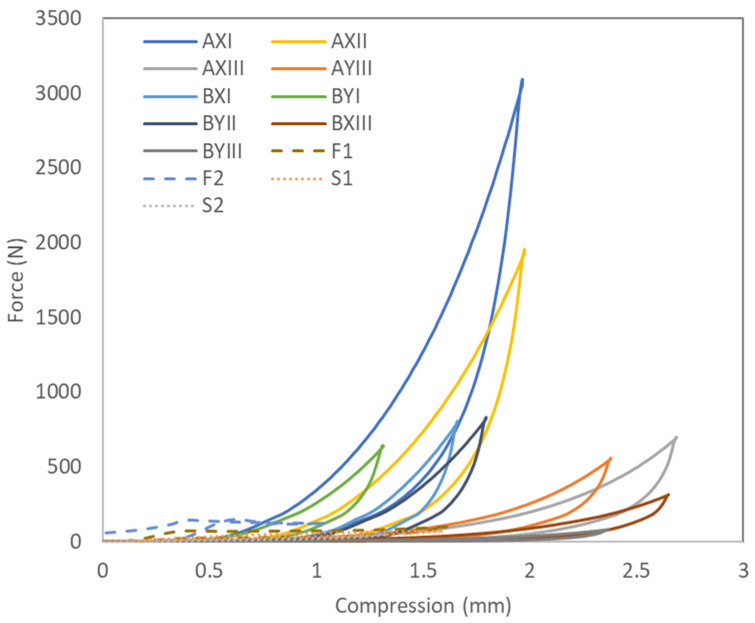
Compression displacement-force curves of fabrics.

In addition, the compression test results in Figure 7 show considerable differences in the mechanical response between the inlay fabric samples and conventional laminated foam or spacer fabrics. The former has a more pronounced plastic region, which indicates a greater ability to withstand plastic deformation before failure. As such, the inlay spacer fabric can withstand higher compression forces before permanent deformation or structural breakdown, exhibiting a higher load-bearing capacity. Such behavior is desirable for materials that need to absorb energy without structural breakdown. Conversely, the laminated foams have almost no plastic regions and rapidly transition to a densified state shortly after the yield point, thus suggesting a lower load-bearing capacity.

The enhanced compression properties of the inlay spacer fabrics can be attributed to the increase in fabric stiffness due to the inlay yarns. Stiffer fabrics distribute and sustain compression forces more effectively and increase load-bearing capacity, thus offering support to the user and reducing the risk of discomfort or injury. This makes inlay spacer fabrics more suitable for applications that require more durability and support, such as protective clothing or cushioning materials.

#### 3.2.4. Force Reduction Performance

**Figure 8:**
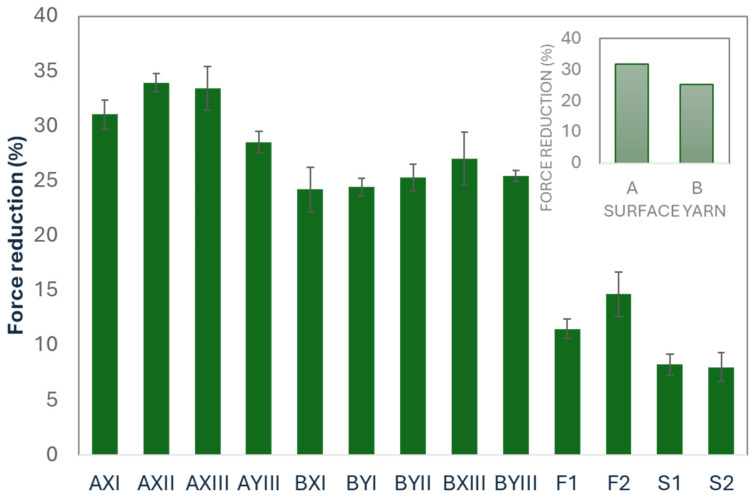
Results of force reduction test.

Force reduction measures the ability of fabrics to absorb and dissipate energy, thereby minimizing the impact force transmitted through them. This property is crucial for applications that require cushioning and impact protection. The results shown in Figure 8 indicate that the percentage reduction in force varies significantly among tested samples (*p* = 0.005), with the inlay samples showing reductions that range from 24–34%, laminated foam samples that range approximately 11–15%, and spacer fabrics of 8%.

The force reduction test results show that the inlay materials have a higher impact reduction capability which implies that they are effective for absorbing energy and providing protection against high-impact forces, which make them ideal for high-impact applications. The inlay yarns and unique construction of the inlay spacer fabrics may enhance the ability of the fabric to absorb energy more effectively than laminated foam and spacer fabrics. The highest force reduction percentage of 33.43% is observed with AXIII.

As shown in Table 6, the type of surface yarn has a statistically significant impact (*p* = 0.009) on the percentage of force reduction. The fabric sample that uses Surface Yarn Type A provides a higher force reduction percentage of 31.7%, while that which uses Surface Yarn Type B is 25.3%. Nevertheless, the type of inlay yarn and pattern type do not show statistically significant differences in the force reduction percentage. Zhang et al. [36] showed that elastic yarns are a key component in the development of high-performance compression garments. In this study, spandex is incorporated into the surface yarn, thereby reducing the force transmitted through the fabrics in protective garments. These findings highlight the importance of selecting the type of surface yarn for fabrics used in applications that require enhanced impact protection.

**Figure 9:**
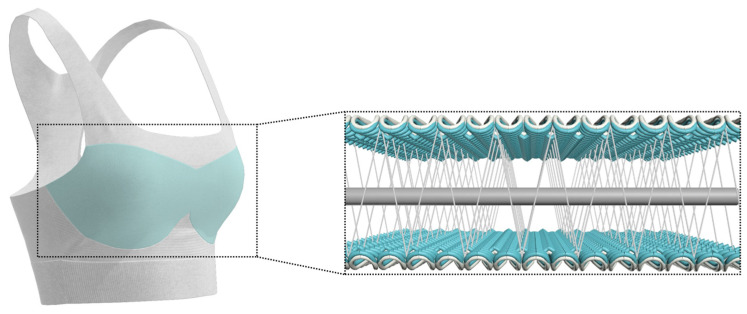
Potential application of inlay fabric as bra pads.

The newly developed inlay fabric has good stretchability, thermal, compression, and force reduction performances, it is recommended to be used in close-fitting apparel that requires cushioning. Figure 9 illustrates the potential application of inlay fabrics in sports bras.

## 4. Conclusions

This study provides a comprehensive analysis of the effects of the inlay structure on the mechanical behavior of newly developed inlay spacer fabrics. To improve the tensile and thermal properties for apparel applications, two surface yarns with superior thermal, surface, and extension behavior are chosen for the fabrication of inlay spacer fabrics. The physical properties of inlay samples are systematically analyzed and compared with those of traditional laminated foam and spacer fabrics. The inlay spacer fabrics show a superior performance in compression, force reduction, and thermal comfort, thus making them ideal for applications that require a high degree of durability and protection from impact. This study further confirms that the type of inlay yarn and the inlay structure play a crucial role in changing fabric properties, including thermal properties and tensile and compressive properties. Specifically, Pattern III has a stretchability comparable to that of conventional fabrics. Surface Yarn A performs well in terms of force reduction, while Pattern I has significant effects on compression performance and thermal conductivity. The results of this study indicate that incorporating a single layer of inlay yarn can effectively enhance both compression performance and thermal conductivity when compared to conventional laminated foams and spacer fabrics while maintaining stretchability, thereby offering a superior alternative in protective clothing applications. This study highlights the potential of inlay knitting techniques in the development of advanced textile structures that balance wear comfort, flexibility, and protection. Future research can focus on optimizing inlay patterns and exploring new yarn combinations to further enhance the performance of these fabrics. It is also recommended that new inlay structures for spacer fabric and different inlay combinations are developed and evaluated. Finally, future research can focus on investigating the pattern of single-layer inlay fabrics without spacer yarns to provide excellent stretchability without affecting other performance characteristics for various applications.

## Figures and Tables

**Table 1:** Single jersey knitting for surface yarn selection.

Yarn Code	Composition	Weight (g/m)	Thread Diameter (mm)	Linear Density (Nm)	Gauge	Twist Type
N1	80% Recycled Viscose 20% Nylon	33.33	0.26	1/30	14	S
N2	60% Organic Cotton 40% Renew Acetate	27.78	0.23	2/36	12	S
N3	30% Merino Wool 70% Cotton	29.41	0.24	2/34	12	Z
N4	30% Merino Wool 35% Viscose 35% Nylon	33.33	0.26	2/30	12	S
N5	77% Acetate 23% Nylon	27.03	0.22	1/37	14	Z
N6	60% Acrylic 40% Cupro	38.46	0.25	2/52	10	S
N7	55% Cotton 45% Polyester	33.33	0.26	2/30	12	S

**Table 2:** Summary of fabric contents.

Parameter	Composition
Surface Yarn A	Surface Yarn A, along with 15%/85% Spandex/Nylon blended yarn
Surface Yarn B	Surface Yarn B, along with 15%/85% Spandex/Nylon blended yarn
Inlay Yarn X	100% Recycled Stretch Nylon
Inlay Yarn Y	100% Recycled Polyester
Pattern I	1 layer of inlay yarns inserted between surface layers by using KN70/70 Lycra as spacer yarn
Pattern II	2 layers of inlay yarns inserted between surface layers by using KN70/70 Lycra as spacer yarn
Pattern III	1 layer of inlay yarns inserted between surface layers, by crossing surface yarns between the front and back needle beds
F1	Laminated polyurethane foam with 100% polyester fabric
F2	Laminated polystyrene foam with 100% polyester fabric
S1	Spacer fabric with 92% polyester and 8% spandex
S2	Spacer fabric with 93% polyester and 7% spandex

**Table 4:** Summary of test methods.

	Test Parameter	Standard	Device
Thermal comfort	Water vapor transmission rate	ASTM E96-22	Water Vapor Permeability Tester
	Thermal conductivity	ASTM F1868	KES Thermo Labo II
Surface	Surface roughness, surface friction	JSA JIS B0601	KES-FB4-A Surface Tester
Stretchability	Load, strain	EN 14704	Instron 5566 Tensile Tester
Compression	Force	ASTM D575	Instron 5566 Tensile Tester
	Force reduction	ASTM D2632	Dynamic load cell

**Table 3:** Inlay fabric design combinations.

Fabric	Surface Yarn	Inlay Yarn	Pattern
AXI	A	X	I
AXII	A	X	II
AXIII	A	X	III
AYIII	A	Y	III
BXI	B	X	I
BYI	B	Y	I
BYII	B	Y	II
BXIII	B	X	III
BYIII	B	Y	III

**Table 5:** Thermal, surface, and extension properties of surface yarns for single jersey knitted structure.

	Thermal Comfort		Surface								Stretchability	
Yarn Code	WVT (g/m^2^/day)	Thermal Conductivity (W/cm·°C)	Surface Friction (MIU)				Surface Roughness (SMD)				Load (N)	
			Wale		Course		Wale		Course		Wale	Course
			Face	Back	Face	Back	Face	Back	Face	Back		
N1	33.03	0.05 4	0.24	0.51	0.42	0.22	3.77	11.22	8.41	2.92	7.65	1.20
N2	31.03	0.047	0.28	0.40	0.29	0.22	2.51	8.48	5.66	3.27	58.61	1.25
N3	31.80	0.045	0.25	0.40	0.30	0.25	7.79	2.58	4.38	2.88	80.44	2.10
N4	36.50	0.043	0.30	0.44	0.36	0.29	2.60	6.71	6.17	2.78	66.48	3.26
N5	32.80	0.045	0.32	0.53	0.45	0.35	3.01	9.59	7.16	2.84	2.19	0.93
N6	32.11	0.047	0.38	0.55	0.36	0.31	4.38	14.32	8.50	2.56	145.54	2.37
N7	32.19	0.0 49	0.36	0.48	0.29	0.25	2.64	9.03	5.93	2.73	81.43	1.16

The two most desirable values are highlighted in shaded cells.

**Table 6:** Effect of using different types of surface and inlay yarns and pattern.

	Surface Yarn	Mean	*p*	Inlay Yarn	Mean	*p*	Pattern	Mean	*p*
Load (N) in wale direction	A	678.0	0.320	X	627.8	0.648	I	580.7	0.067
	B	539.2		Y	567.3		II	880.1	
							III	476.4	
Load (N) in course direction	A	17.4	0.183	X	16.0	0.493	I	14.4	0.429
	B	13.3		Y	14.1		II	20.2	
							III	13.1	
WVT (g/m²/day)	A	561.3	0.093	X	586.2	0.494	I	616.0	0.645
	B	627.6		Y	613.1		II	565.5	
							III	601.1	
Thermal conductivity (W/m·K)	A	0.04	0.898	X	0.04	0.447	I	0.06	0.026
	B	0.04		Y	0.04		II	0.05	
							III	0.02	
Force (N)	A	1542.2	0.177	X	1342.7	0.177	I	1477.3	0.375
	B	520.0		Y	513.9		II	1360.7	
							III	403.9	
Force reduction (%)	A	31.7	0.009	X	29.9	0.107	I	26.6	0.788
	B	25.3		Y	25.9		II	29.6	
							III	28.6	

Significant values are highlighted in shaded cells.

**Table 7:** Result of mean value and one-way ANOVA between inlay samples, laminated foam, and spacer fabric.

			Thermal Comfort		Compression		Stretchability	
	Fabric Code	Thickness (mm)	WVT (g/m^2^ day)	Thermal Conductivity (W/m·K)	Force (N)	Force Reduction (%)	Load (N)	
							Wale	Course
Inlay fabric	AXI	3.9	556.2	0.064	3028.2	31.05	728.6	15.2
	AXII	3.9	530.4	0.054	1910.7	33.95	951.7	24.0
	AXIII	5.3	521.1	0.025	684.3	33.43	539.6	13.7
	AYIII	4.7	637.6	0.022	545.5	28.55	492.1	16.6
	BXI	3.3	643.1	0.054	781.5	24.22	539.3	15.8
	BYI	3.2	648.6	0.047	622.3	24.44	474.1	12.3
	BYII	3.6	600.6	0.048	810.7	25.31	808.6	16.4
	BXIII	5.3	680.1	0.024	308.7	26.99	379.7	11.1
	BYIII	4.7	565.5	0.025	76.9	25.44	494.2	11.0
Mean		4.2	598.1	0.04	974.3	28.15	600.9	15.1
Laminated foam	F1	3.2	571.0	0.006	98.7	11.49	63.7	14.8
	F2	3.4	648.6	0.032	148.2	14.66	70.9	17.2
Mean		3.3	609.8	0.019	123.4	13.08	67.3	16.0
Spacer fabric	S1	3.1	584.0	0.017	46.3	8.25	425.6	6.9
	S2	3.2	541.5	0.016	71.6	8.02	447.2	31.6
Mean		3.1	562.7	0.017	58.9	8.13	436.4	19.2
*p*-value		0.069	0.557	0.094	0.081	0.005	<0.001	0.888

Significant values are highlighted in shaded cells.

## Data Availability

The data presented in this study are available upon request from the corresponding author due to ethical reasons.
